# NAD⁺ biology and supplementation: From mechanisms to clinical perspectives

**DOI:** 10.1007/s11033-026-12351-3

**Published:** 2026-07-23

**Authors:** Sergio Pandolfi, Charlye Ghezzi, Geir Björklund, Marco Metalla, Francesco Maria Paone, Salvatore Chirumbolo

**Affiliations:** 1 High Master School of Oxygen-Ozone Therapy, Section Neurosurgery, University of Pavia, Pavia, Italy; 2 Council for Nutritional and Environmental Medicine, Mo i Rana, Norway; 3 University of Tor Vergata, Rome, Italy; 4 Department of Engineering for Innovation Medicine, University of Verona, Strada Le Grazie 8, Verona, 37134 Italy

**Keywords:** NAD, Nutraceuticals, Pharmacokinetics, Infusion, Doses

## Abstract

This study examines the biological and clinical relevance of NAD⁺ supplementation using a combined review and mathematical modelling approach. NAD⁺ plays a central role in cellular energy metabolism, redox balance, and signaling pathways linked to aging, neurodegeneration, and metabolic health. Current evidence shows that oral NAD⁺ precursors such as nicotinamide riboside and nicotinamide mononucleotide can increase circulating NAD⁺ levels, although their clinical benefits remain variable and context-dependent. Intravenous NAD⁺ administration is less well characterized and lacks robust clinical validation. The modelling framework presented here highlights that NAD⁺ responses are nonlinear and influenced by factors such as dose, age, metabolic state, and route of administration. Rather than following a simple dose–response relationship, NAD⁺ supplementation appears to operate within a complex regulatory system involving feedback mechanisms and biological saturation. Overall, these findings emphasize the need for cautious interpretation of current data and for well-designed clinical studies to define effective and safe therapeutic strategies.

## Introduction

Nicotinamide adenine dinucleotide (NAD+) and its reduced form NADH, together with the phosphorylated redox pair NADP+/NADPH, are essential cofactors that coordinate cellular energy metabolism, mitochondrial oxidative phosphorylation, antioxidant defense, DNA repair, and redox-sensitive signaling in the brain, thereby influencing neuronal survival and vulnerability during aging and neurodegenerative processes [1–4]. Their importance extends beyond basic bioenergetics into the regulation of neuronal survival, synaptic plasticity, oxidative stress responses, and gene expression, all of which are critical processes underlying cognition, mood regulation, and the pathophysiology of neuropsychiatric and neurodegenerative disorders [5–10].

Over the past decades, evidence from molecular, neurochemical, preclinical, and clinical studies has increasingly shown that disruption of NAD+ metabolism and NAD+/NADH and NADP+/NADPH redox balance is associated with impaired mitochondrial function, oxidative stress, neuroinflammation, cognitive decline, depression-related phenotypes, and neurodegenerative diseases, including Alzheimer’s disease and Parkinson’s disease [2, 5, 8, 11, 12].

NAD⁺ functions primarily as an electron carrier in redox reactions, cycling between oxidized (NAD⁺) and reduced (NADH) states in metabolic pathways such as glycolysis, the tricarboxylic acid cycle, and oxidative phosphorylation. In neurons, which have exceptionally high energy demands, the NAD⁺/NADH ratio is crucial for maintaining mitochondrial function and ATP production [13–16].

A decline in NAD⁺ availability impairs mitochondrial respiration and promotes the accumulation of reactive oxygen species, leading to oxidative stress and neuronal damage. Studies such as those by Verdin (2015) and Ying (2008) have emphasized that NAD⁺ depletion is a hallmark of aging and is exacerbated in neurodegenerative diseases, contributing to impaired neuronal resilience and synaptic dysfunction [17–22].

This evidence would support the suggestion that NAD^+^ supplementation, even by venipuncture (IV), may address depletion concern.

Beyond its metabolic role, NAD⁺ serves as a substrate for several classes of enzymes involved in cellular signaling, including sirtuins, poly-(ADP-ribose) polymerases (PARPs), and CD38. Sirtuins, particularly SIRT1 and SIRT3, are NAD⁺-dependent deacetylases that regulate gene expression, mitochondrial biogenesis, and stress resistance. Activation of sirtuins has been associated with improved cognitive function and neuroprotection in animal models [23–26].

Research by Herskovits and Guarente (2014) demonstrated that enhancing NAD⁺ levels can stimulate SIRT1 activity, which in turn promotes synaptic plasticity and protects against neurodegeneration [23]. Conversely, excessive activation of PARPs during DNA damage can deplete cellular NAD⁺ pools, leading to energy failure and cell death, a mechanism implicated in stroke and neurodegenerative pathology [24–29].

The NADP⁺/NADPH system plays a complementary yet distinct role, primarily in anabolic reactions and antioxidant defense. NADPH provides reducing power for biosynthetic processes and is essential for maintaining glutathione in its reduced form, which is one of the brain antioxidants. Oxidative stress is a well-established contributor to depression and neurodegenerative diseases, and impaired NADPH production can compromise the brain ability to neutralize reactive oxygen species. Research by Mailloux et al. (2013) and others has shown that disturbances in NADPH-generating pathways, such as the pentose phosphate pathway, are linked to increased oxidative damage in neuronal cells [30–35].

In the context of cognition, NAD⁺ availability has been directly associated with synaptic function and memory formation. Experimental studies have demonstrated that boosting NAD⁺ levels through precursors such as nicotinamide riboside (NR) or nicotinamide mononucleotide (NMN) enhances learning and memory in animal models. For example, Gong et al. (2013) found that NAD⁺ supplementation improved cognitive performance in mice by enhancing mitochondrial function and reducing neuroinflammation. These findings suggest that NAD⁺ depletion may contribute to age-related cognitive decline and that restoring NAD⁺ levels could be a potential therapeutic strategy [36].

Depression and mood disorders have also been linked to alterations in NAD⁺ metabolism and redox balance. Chronic stress, a major risk factor for depression, has been shown to disrupt mitochondrial function and reduce NAD⁺ levels in the brain [37, 38]. This can lead to impaired energy metabolism and increased oxidative stress, both of which are observed in patients with major depressive disorder. Furthermore, inflammation, which is increasingly recognized as a contributor to depression, can activate enzymes such as indoleamine 2,3-dioxygenase (IDO) that divert tryptophan metabolism toward the kynurenine pathway, producing neurotoxic metabolites and further affecting NAD⁺ synthesis. Studies such as those by Otte et al. (2016) highlight the interplay between metabolic, inflammatory, and neurochemical pathways in depression, with NAD⁺ serving as a central node [39–41].

In neurodegenerative diseases, the role of NAD⁺ and NADPH becomes even more pronounced. In Alzheimer’s disease, reduced NAD⁺ levels have been associated with mitochondrial dysfunction, amyloid-beta accumulation, and tau pathology. Hou et al. (2018) demonstrated that increasing NAD⁺ levels in mouse models of Alzheimer’s disease improved cognitive function and reduced neurodegeneration, partly through activation of SIRT1 and enhancement of DNA repair mechanisms. Similarly, in Parkinson’s disease, NAD⁺ depletion contributes to dopaminergic neuron vulnerability, and strategies aimed at restoring NAD⁺ levels have shown neuroprotective effects in preclinical studies.

Another important aspect is the involvement of NAD⁺ in circadian rhythm regulation, which has implications for mood and cognitive function. NAD⁺ levels oscillate in a circadian manner and regulate the activity of clock genes through sirtuin-mediated deacetylation. Disruption of circadian rhythms is commonly observed in depression and neurodegenerative diseases, suggesting that NAD⁺ metabolism may link metabolic state to behavioural and psychological outcomes [37–42].

Clinical interest in NAD⁺-boosting interventions has grown significantly, with ongoing trials investigating the efficacy of NAD⁺ precursors in improving cognitive function and mood. While early results are promising, challenges remain in understanding the optimal dosing, bioavailability, and long-term effects of such interventions. Additionally, the complexity of NAD⁺ metabolism, which involves multiple biosynthetic and salvage pathways, requires a nuanced approach to therapeutic targeting.

In summary, NAD⁺/NADH and NADP⁺/NADPH systems are fundamental to brain function, influencing energy metabolism, oxidative stress, and cellular signaling pathways that underpin cognition and emotional regulation. Disruptions in these systems are closely linked to depression, neurodegenerative diseases, and mood disorders, making them important targets for future research and therapeutic development. The growing body of evidence underscores the need for a deeper understanding of NAD⁺ biology in the brain, as well as the translation of these insights into effective clinical interventions.

A major unresolved issue concerns dosing. Both oral and IV NAD⁺ strategies require careful evaluation, because dose, route, frequency, bioavailability, safety, and clinical efficacy may differ substantially. Defining appropriate dose ranges for oral precursors and IV NAD⁺ remains essential before therapeutic recommendations can be made.

This review tries to add a fundamental contribution for a thorough elucidation of the role of NAD^+^/NADH in these topics.

## Literature search

A literature search using the terms “NAD [AND] clinical [AND] efficacy,” (1,005 items in PubMED), “NAD [AND] supplementation,” (1,373 items in PubMED) “NAD [AND] cognition,” (578 items on PubMED) and “NAD [AND] neurodegenerative disorder” (1,605 items on PubMED) across PubMed, Scopus, and Web of Science identifies a fast-growing but still immature clinical field. The strongest and most consistent signal is biochemical rather than therapeutic: NAD⁺ precursors, especially NR and NMN, repeatedly increase circulating NAD⁺ or NAD-related metabolites in humans. However, the translation of this biochemical effect into robust clinical efficacy remains variable. PubMed-indexed clinical studies show that NAD⁺ augmentation has been explored in metabolic disease, mitochondrial disorders, neurodegeneration, cognitive impairment, cardiovascular disease, inflammatory disease, and post-viral syndromes. The clearest disease-specific evidence comes from mitochondrial myopathy, where niacin corrected systemic NAD⁺ deficiency and improved muscle performance, suggesting that patients with demonstrable NAD⁺ deficiency may be especially responsive. In metabolic studies, NMN improved muscle insulin sensitivity in postmenopausal women with prediabetes, while other NMN trials reported increased blood NAD⁺ and modest physical-performance signals, but not broad metabolic transformation.

The search terms related to cognition and neurodegeneration retrieve a smaller, more mechanistic literature. The NADPARK trial in Parkinson’s disease showed that nicotinamide riboside enhanced cerebral and systemic NAD metabolome signatures, but was not designed to prove disease modification. In mild cognitive impairment, nicotinamide riboside increased blood NAD⁺ concentrations but did not clearly improve cognition in the randomized trial reported by Orr et al. [6]. Other studies using neuron-derived extracellular vesicles suggest that oral NR can increase neuronal NAD⁺-related biomarkers and reduce markers linked to amyloid and stress-kinase signaling, but these remain biomarker rather than definitive cognitive outcomes. Acute magnetic resonance spectroscopy studies further support the biological plausibility that NR can raise brain NAD⁺ in humans.

Across Scopus and Web of Science, the broader pattern is similar: many reviews and preclinical papers, fewer adequately powered randomized clinical trials, and substantial heterogeneity in compound, dose, duration, population, and endpoints. Recent systematic reviews conclude that NAD⁺ supplementation is promising but not yet proven as a general anti-aging or neuroprotective therapy. Overall, the literature supports target engagement and safety, while clinical efficacy remains condition-specific and incompletely established.

### Insights on recent preclinical studies

Table 1A shows the most recent preclinical studies regarding NAD^+^/NADH.

NAD⁺ preclinical research from 2020 onward has progressively moved beyond the simplistic idea of NAD⁺ as a generic anti-aging metabolite and toward a more refined view of NAD⁺ as a dynamic regulator of cellular resilience, metabolic adaptation, inflammation, and tissue-specific vulnerability. The studies summarized in Table 1A show that NAD⁺ biology is not governed only by precursor availability, but by the balance between biosynthesis, enzymatic consumption, tissue uptake, inflammatory state, mitochondrial demand, and disease context. This is an important conceptual evolution: NAD⁺ decline is increasingly interpreted not merely as a passive consequence of aging, but as an active biological node capable of amplifying mitochondrial dysfunction, sterile inflammation, impaired repair, and loss of tissue homeostasis [43–60].

A central mechanistic contribution comes from studies on CD38, one of the major NAD⁺-consuming enzymes. Chini et al. showed that aging is associated with induction of CD38-positive immune cells in tissues such as white adipose tissue and liver, linking inflammaging to tissue NAD⁺ depletion [43]. This finding is highly relevant because it reframes age-related NAD⁺ loss as partly driven by inflammatory NADase activity rather than only by reduced biosynthesis. In this sense, NAD⁺ decline becomes a consequence of altered tissue ecology: senescent and inflammatory cells consume NAD⁺ and thereby weaken metabolic function in surrounding tissues [43].

The later work on CD38 inhibition with 78c further supports this model, showing that pharmacological reduction of NAD⁺ consumption can improve healthspan and lifespan-related outcomes in aged mice [48]. Together, these studies suggest that preserving NAD⁺ may require not only supplementation with precursors such as NR or NMN, but also inhibition of excessive NAD⁺ degradation.

The reproductive aging studies provide one of the clearest examples of functional NAD⁺ rescue in a highly specialized cell type. Miao et al. demonstrated that NMN supplementation restored NAD⁺ levels in aged mouse oocytes and improved ovulated oocyte number, meiotic competence, fertilization ability, mitochondrial function, and embryo developmental potential [44]. Subsequent studies reinforced this observation, showing that NMN can improve mitochondrial membrane potential and developmental competence in aged oocytes [51], while NR can protect against postovulatory aging during in vitro culture [54]. These findings are important because oocytes are exquisitely dependent on mitochondrial integrity and redox balance. NAD⁺ repletion therefore appears to act not simply as metabolic “fuel,” but as a stabilizer of cellular quality control during reproductive aging.

Kidney injury models provide another strong mechanistic framework. Doke et al. showed that NAD⁺ deficiency during acute kidney injury promotes mitochondrial dysfunction, cytosolic mitochondrial RNA release, and RIG-I–dependent inflammatory activation, while NR or NMN supplementation restored NAD⁺ and improved kidney injury markers [50]. This study is particularly elegant because it connects NAD⁺ depletion to innate immune activation through mitochondrial danger signaling. In other words, NAD⁺ insufficiency does not merely impair energy metabolism; it can convert mitochondrial stress into inflammatory pathology. More recent chronic kidney disease models extend this idea, suggesting that NAD⁺ repletion may activate renal sirtuin signaling and protect against progressive renal damage [57]. These studies position NAD⁺ as a mediator between mitochondrial health and organ-level inflammatory injury.

Metabolic liver disease studies also support the idea that NAD⁺ modulation can remodel tissue metabolism. NMN administration in aged mouse liver altered the hepatic protein acetylome, consistent with modulation of NAD⁺-dependent deacetylases such as sirtuins [48]. Similarly, integrated transcriptomic and metabolomic analyses in NAFLD models reported that NR and NMN influence lipid metabolism, inflammatory pathways, and mitochondrial function [53].

However, the table also rightly includes negative or modest studies, such as Cartwright et al., who found that NR had minimal impact on energy metabolism in mouse models of mild obesity [45]. This is crucial for scientific balance. NAD⁺ precursors are not universal metabolic correctors; their effects depend on disease severity, tissue demand, genetic background, dose, duration, and the enzymatic machinery available to convert precursors into NAD⁺.

The pharmacology of NAD⁺ precursors is itself more complex than initially assumed. Kropotov et al. identified purine nucleoside phosphorylase as a regulator of NR metabolism and NAD⁺ synthesis [47]. This matters because it challenges the assumption that oral or systemic administration of an NAD⁺ precursor automatically produces equivalent NAD⁺ increases across tissues. Tissue-specific enzyme expression, precursor routing, and metabolic competition all shape biological response. This helps explain why some preclinical studies show strong benefits while others report modest or context-dependent effects.

In neurodegenerative and retinal models, NAD⁺ repletion appears particularly relevant because neurons and retinal ganglion cells are highly dependent on mitochondrial homeostasis. Zhang et al. showed that systemic NR protected retinal ganglion cells in mouse models of optic nerve crush and ocular hypertension, preserving function and reducing inflammatory markers [46]. More recent neurodegeneration studies using NMN similarly reported improvements in mitochondrial dysfunction and neuronal injury phenotypes [55]. These models support the broader view that NAD⁺ is especially important in long-lived, energetically demanding cells where mitochondrial failure has irreversible consequences.

Inflammation-focused models further strengthen the immunometabolic role of NAD⁺.

Ahmed et al. reported that NMN restored NAD⁺ levels and reduced inflammatory and oxidative stress endpoints in an LPS-induced inflammatory model [56]. This aligns with the CD38 and kidney injury studies, suggesting that NAD⁺ depletion and inflammation may reinforce each other in a pathological loop: inflammation increases NAD⁺ consumption, and NAD⁺ loss worsens mitochondrial dysfunction and inflammatory signaling.

At the same time, the table appropriately emphasizes caution in oncology. Cancer biology represents a major exception to simplistic NAD⁺-boosting narratives. Many tumours rely on NAD⁺ metabolism to support proliferation, DNA repair, redox balance, and metabolic plasticity. Therefore, in selected cancer contexts, inhibition of NAMPT or other NAD⁺ biosynthetic pathways may be therapeutically useful rather than harmful [60]. This point is essential: NAD⁺ is not intrinsically “good” or “bad”; its biological meaning depends on cellular context. In degenerative tissues, NAD⁺ restoration may enhance repair and resilience, whereas in malignant cells, NAD⁺ availability may support survival and growth.

Overall, the recent preclinical literature supports a nuanced conclusion. NAD⁺ modulation has substantial therapeutic promise in aging biology, reproductive decline, kidney injury, liver metabolism, neurodegeneration, retinal disease, and inflammatory stress. Yet the strongest interpretation is not that NAD⁺ boosters are universally beneficial, but that NAD⁺ metabolism is a controllable systems-level regulator of tissue function. The most rigorous future strategies will likely combine precursor supplementation, inhibition of excessive NAD⁺ consumption, tissue-specific delivery, biomarker-guided dosing, and careful exclusion of contexts where NAD⁺ enhancement could be harmful, particularly cancer or high-proliferation states.

### Insights on recent clinical studies

Table 2 shows the most recent clinical studies regarding NAD^+^/NADH.

**Table 1:** Important Preclinical NAD⁺ Studies (2020–April 2026)

Year	Study	Model/system	Intervention or NAD⁺ mechanism	Domain	Main preclinical finding	Why it matters	Ref.
2020	Chini et al., Nat Metab	Aged mice; immune-cell/senescent-cell models	Mechanistic manipulation of CD38/NADase biology	Aging, inflammaging, NAD+ decline	Showed age-associated induction of CD38 + immune cells in white adipose tissue and liver and linked CD38 to tissue NAD+ decline and altered NMN/NAD metabolism.	Key mechanistic paper: age-related NAD+ depletion is not only reduced synthesis; increased consumption by CD38 is central.	[43]
	CD38 ecto-enzyme in immune cells is induced during aging and regulates NAD + and NMN levels						
2020	Miao et al., Cell Reports	Naturally aged female mice; oocytes/embryos	NMN supplementation	Reproductive aging	Restored NAD + in aged oocytes, improved ovulated oocyte number, meiotic competence, fertilization ability, mitochondrial function, and embryo development potential.	Important early 2020s study linking NAD+ repletion to female reproductive aging phenotypes.	[44]
	Nicotinamide Mononucleotide Supplementation Reverses the Declining Quality of Maternally Aged Oocytes						
2021	Cartwright et al., J Endocrinol	Mouse models of mild obesity; strain-comparison approach	Dietary nicotinamide riboside (NR)	Metabolism/obesity	NR effects were modest and context dependent; the study challenged broad claims that NR universally improves metabolic endpoints in obesity models.	Important negative/neutral preclinical study; helps avoid publication-bias overinterpretation of NAD+ boosters.	[45]
	Nicotinamide riboside has minimal impact on energy metabolism in mouse models of mild obesity						
2021	Zhang et al., Pharmaceutics	Mouse optic nerve crush and microbead-induced ocular hypertension	Systemic NR	Glaucoma/retinal neurodegeneration	NR enhanced retinal ganglion cell survival, preserved pattern ERG function in the acute injury model, and suppressed retinal inflammation markers.	Strong preclinical rationale for NAD+ support in retinal ganglion cell vulnerability.	[46]
	Systemic Treatment with Nicotinamide Riboside Is Protective in Two Mouse Models of Retinal Ganglion Cell Damage						
2022	Kropotov et al., Nat Commun	Human cells and mouse tissues	Genetic/pharmacologic analysis of PNP in NR metabolism	NAD+ precursor pharmacology	Identified purine nucleoside phosphorylase as a regulator of NR metabolism and tissue NAD+ boosting, refining assumptions about NR bioavailability and conversion.	Important mechanistic pharmacology study: precursor efficacy depends on tissue metabolism and enzyme context.	[47]
	Purine nucleoside phosphorylase controls nicotinamide riboside metabolism and NAD+ synthesis						
2022	Luo et al., Front Aging? / aging liver proteomics	Aged mouse liver; acetylome/proteome analysis	NMN administration	Aging liver, sirtuin-linked acetylation	Reported broad changes in liver protein acetylation patterns after NMN, consistent with modulation of NAD+-dependent deacetylase biology.	Useful omics-level preclinical evidence that NAD+ repletion can remodel age-associated protein acetylation.	[48]
	Nicotinamide Mononucleotide Administration Amends Protein Acetylome of Aged Mouse Liver						
2022	Peclat et al., Aging Cell	Chronologically aged mice	Small-molecule CD38 inhibitor 78c	Aging, healthspan, NAD+ consumption	Reported lifespan/healthspan benefits in aged mice with pharmacologic CD38 inhibition, supporting NAD+ preservation via reduced consumption.	Important because it targets NAD+ degradation rather than only adding precursors.	[49]
	CD38 inhibitor 78c increases mice lifespan and healthspan in a model of chronological aging						
2023	Doke et al., Nat Metab	Male mouse cisplatin- and ischemia–reperfusion kidney injury models; renal tubular cells	NR or NMN supplementation	Acute kidney injury/inflammation	NAD+ deficiency promoted mitochondrial dysfunction, cytosolic mtRNA release and RIG-I inflammatory activation; NR/NMN restored NAD + and improved kidney function markers.	High-impact mechanistic study connecting NAD+ loss to innate immune activation in organ injury.	[50]
	NAD+ precursor supplementation prevents mtRNA/RIG-I-dependent inflammation during kidney injury						
2023	Li et al., Reprod Biol Endocrinol	Aged mouse oocytes; in vitro maturation/embryo-development assays	NMN treatment	Reproductive aging	NMN improved mitochondrial membrane potential and developmental competence of aged oocytes.	Independent reinforcement of NAD+ precursor effects on oocyte quality; still preclinical.	[51]
	β−Νιχοτιναµιδε Μονονυχλεοτιδε ρεσχυεσ τηε θυαλιτψ οφ αγεδ οοχψτεσ						
2024	Malique et al., Sci Transl Med	Preclinical mouse/environmental-enteric-dysfunction models	NAD+ precursors with bile-acid sequestration strategy	Environmental enteric dysfunction, malnutrition, gut biology	Linked dysregulated bile-acid signaling and NAD+ synthesis to EED pathophysiology and showed therapeutic benefit in preclinical models.	Important translational NAD+ study outside classic aging/metabolism fields.	[52]
	NAD+ precursors and bile acid sequestration treat preclinical refractory environmental enteric dysfunction						
2024	Zhang & Chen, Biomed Pharmacother	NAFLD preclinical model with transcriptome/metabolome profiling	NR and NMN	NAFLD/metabolic liver disease	Reported multi-omic remodeling consistent with improvement of NAFLD-related metabolic and inflammatory pathways after NAD+ precursor treatment.	Useful comparative preclinical study of NR vs. NMN in liver-metabolic disease.	[53]
	Integrated transcriptome and metabolome study reveals therapeutic effects of NR and NMN on nonalcoholic fatty liver disease						
2024	Li et al., Front Endocrinol? / ovarian aging	Mouse oocytes undergoing postovulatory aging in vitro	NR supplementation	Oocyte quality/reproductive biology	NR reduced postovulatory-aging phenotypes and supported oocyte quality during culture.	Expands reproductive NAD+ literature from NMN to NR, but mostly ex vivo/in vitro preclinical evidence.	[54]
	The NAD+ precursor nicotinamide riboside protects against postovulatory aging during in vitro culture						
2024	Xiong et al., Cell Death Dis	Preclinical neurodegeneration model; neuronal/mitochondrial assays	NMN	Neurodegeneration	Reported neuroprotective and mitochondrial benefits from NMN-mediated NAD+ boosting in disease-relevant models.	Representative of the growing 2024 neurodegeneration literature; translation remains unproven.	[55]
	NAD+-boosting agent nicotinamide mononucleotide ameliorates neurodegeneration and mitochondrial dysfunction						
2024	Ahmed et al., Antioxidants	LPS inflammatory model; immune/cellular assays	NMN	Inflammation/sepsis-like stress	Reported restoration of NAD + and attenuation of inflammatory/oxidative stress endpoints after NMN.	Supports NAD + as an immunometabolic node; model-specific and not direct human efficacy evidence.	[56]
	Nicotinamide Mononucleotide Restores NAD+ Levels to Alleviate LPS-Induced Inflammation						
2025	Jones et al., JCI Insight? / renal model	Mouse model of Alport syndrome/chronic kidney disease	NR/NAD+ repletion strategy	Chronic kidney disease	Reported kidney-protective effects through NAD+-linked renal sirtuin/mitochondrial biology in a chronic disease model.	Extends NAD+ repletion beyond acute injury into chronic renal disease models.	[57]
	NAD+ prevents chronic kidney disease by activating renal sirtuin signaling						
2025	Liang et al., ovarian senescence model	Mouse ovarian/granulosa-cell senescence model	NMN	Ovarian aging/senescence	NMN improved cellular senescence markers and mitochondrial/oxidative-stress phenotypes.	Further supports reproductive-aging hypothesis, but remains mechanistic/preclinical.	[58]
	Nicotinamide Mononucleotide improves senescence phenotypes in mouse ovarian cells						
2025	Zhu et al., ovarian dysfunction model	Preclinical ovarian dysfunction model	NR	Ovarian/metabolic reproductive dysfunction	NR improved ovarian and metabolic phenotypes in a disease-relevant preclinical model.	Shows both NR and NMN are being tested in reproductive/ovarian aging contexts.	[59]
	Nicotinamide riboside supplementation ameliorates ovarian dysfunction						
2025	Yusri et al., Nat Cancer Rev? / cancer preclinical synthesis	Cancer preclinical literature synthesis and mechanistic models	NAMPT/NAD+ pathway inhibition or modulation	Cancer metabolism	Summarizes evidence that many cancers depend on NAD+ metabolism and that NAD+ pathway inhibition can be therapeutic in selected models.	Cautionary counterpoint: NAD+ boosting is not universally beneficial; in cancer, NAD+ metabolism may need inhibition, not augmentation.	[60]
	Targeting NAD+ metabolism: preclinical insights into potential cancer therapy strategies						

Scope note: This is a curated evidence map, not an exhaustive systematic review. It deliberately includes positive, neutral, mechanistic, and cautionary studies

Interpretation warning. Preclinical effects do not establish human benefit, dose equivalence, long-term safety, or disease-prevention claims. Cancer and high-proliferation settings require special caution because NAD⁺ metabolism can support tumor growth in some contexts, while NAD⁺ pathway inhibition may be therapeutic in selected cancer models

**Table 2:** Clinical studies of NAD+ boosting and NAD+ supplementation, 2020–April 2026

Year	Study	Design	Population	Intervention	Duration	NAD+ / NAD metabolome effect	Clinical / biological outcome	Interpretation	Reference
2020	Pirinen et al., Cell Metabolism — “Niacin cures systemic NAD+ deficiency and improves muscle performance in adult-onset mitochondrial myopathy.”	Clinical interventional study in adult-onset mitochondrial myopathy; mechanistic tissue/blood NAD+ profiling.	Patients with adult-onset mitochondrial myopathy plus controls; rare-disease cohort.	Escalating high-dose niacin / nicotinic acid.	Up to 10 months reported.	Rescued systemic and muscle NAD+ deficiency.	Improved muscle strength/performance signals and mitochondrial myopathy phenotype measures.	Important disease-targeted NAD+ repletion proof-of-concept; not general anti-aging evidence.	[61]
2021	Yoshino et al., Science — NMN in postmenopausal women with prediabetes.	Randomized, placebo-controlled clinical trial.	25 postmenopausal women with overweight/obesity and prediabetes.	Nicotinamide mononucleotide (NMN), 250 mg/day.	10 weeks.	Increased NAD+ metabolism in muscle-related pathways.	Improved muscle insulin sensitivity, insulin signaling, and skeletal-muscle remodeling; no major body-composition change.	One of the strongest human metabolic NMN trials; sex- and phenotype-specific.	[62]
2021	Altay et al., Advanced Science — combined metabolic activators in mild-to-moderate COVID-19.	Open-label phase 2 plus randomized double-blind phase 3 trials.	Adults with PCR-confirmed mild-to-moderate COVID-19.	Combined metabolic activators including nicotinamide riboside (NR), L-serine, N-acetylcysteine, and L-carnitine tartrate.	14 days.	Aimed at NAD+/glutathione/mitochondrial support; not an isolated NR-only test.	Reported faster symptom-free recovery versus control/placebo.	Clinically interesting but attribution to NAD+ boosting alone is impossible because intervention was multi-compound.	[63]
2021	Zeybel et al., Molecular Systems Biology — combined metabolic activators in NAFLD.	Randomized, placebo-controlled clinical trial.	Patients with nonalcoholic fatty liver disease.	Combined metabolic activators including an NAD+ precursor with serine, NAC, and carnitine support.	Short-term treatment phase.	Targeted NAD + and mitochondrial cofactor networks rather than NAD+ alone.	Reduced liver fat and shifted multi-omics signatures toward improved mitochondrial/fatty-acid oxidation pathways.	Useful systems-medicine evidence; not a clean single-agent NAD+ precursor trial.	[64]
2022	Brakedal et al., Cell Metabolism — NADPARK study in Parkinson’s disease.	Randomized, double-blind phase I trial.	Patients with Parkinson’s disease.	Nicotinamide riboside.	Approximately 30 days.	Enhanced cerebral and systemic NAD metabolome signatures.	Signals of altered brain metabolism and inflammatory markers; exploratory clinical signals only.	Important neurologic proof-of-mechanism; underpowered for disease-modifying conclusions.	[65]
2022	Wang et al., JACC Basic to Translational Science — NR in heart failure.	Open-label dose-escalation/safety study.	Ambulatory stage C heart-failure patients.	Nicotinamide riboside up to 2 g/day.	12 weeks.	Significantly increased whole-blood NAD+ levels.	Safe and tolerated; exploratory inflammatory/functional endpoints, not definitive efficacy.	Key cardiovascular safety and target-engagement study; needs larger RCT efficacy trials.	[66]
2022	Jensen et al., JCI Insight — NR + pterostilbene in experimental muscle injury.	Randomized, double-blind, placebo-controlled trial.	32 older adults, 55–80 years.	NR 1,000 mg/day + pterostilbene 200 mg/day.	Started 14 days before induced muscle injury and continued through recovery.	Increased whole-blood NAD + and related metabolites; no clear skeletal-muscle NAD+ augmentation from supplementation.	No improvement in muscle stem-cell recruitment, muscle recovery, or regeneration endpoints.	Important negative/neutral translational study; blood NAD+ response did not guarantee muscle efficacy.	[67]
2022	Yi et al., GeroScience — β-NMN dose-response in healthy middle-aged adults.	Randomized, multicenter, double-blind, placebo-controlled parallel-group trial.	Healthy middle-aged adults.	β−ΝΜΝ 300, 600, ορ 900 µγ/δαψ.	60 days.	Dose-dependent increase in blood NAD concentrations.	Generally safe and well tolerated; improvements in some health/physical-performance related measures; no HOMA-IR difference.	Major NMN safety/biomarker trial, but clinical endpoints remain mostly exploratory.	[68]
2022	Igarashi et al., NPJ Aging — chronic NMN in older men.	Randomized, placebo-controlled trial.	Healthy older men.	NMN 250 mg/day.	12 weeks.	Reported increase in NAD+ metabolites.	Improved some lower-limb physical-performance measures; insulin sensitivity and fat mass unaffected.	Suggestive geroscience signal; small sample and limited clinical scope.	[69]
2022	Fukamizu et al., Scientific Reports — high-dose β-NMN safety.	Clinical safety/tolerability study.	Healthy adult men and women.	β−ΝΜΝ 1,250 µγ ονχε δαιλψ.	4 weeks.	Safety-focused; NAD+ pharmacodynamic detail limited compared with efficacy trials.	Reported safe and well tolerated at the tested dose.	Useful upper-dose tolerability evidence; not designed to prove efficacy.	[70]
2022	Vreones et al., Aging Cell — oral NR and neuron-derived extracellular vesicle biomarkers.	Clinical biomarker study in older adults with mild cognitive impairment.	Older adults with MCI.	Oral nicotinamide riboside.	Short supplementation period.	Increased NAD+ levels in neuron-derived extracellular vesicles.	Reduced EV biomarkers linked to amyloid and stress-kinase pathways; clinical cognition not definitive.	Important brain-access/biomarker study; hypothesis-generating.	[71]
2023	Lapatto et al., Science Advances / related report — NR in monozygotic twin pairs.	Placebo-controlled twin study.	Monozygotic twins.	Nicotinamide riboside.	5 months.	Increased systemic NAD+ metabolites.	Reported changes in muscle mitochondrial biogenesis, myoblast differentiation, and gut microbiota; functional outcomes limited.	Mechanistically rich but not definitive clinical efficacy evidence.	[72]
2023	Dellinger et al., Hepatology — NR + pterostilbene in NAFLD.	Double-blind, placebo-controlled clinical trial.	Patients with NAFLD.	NR plus pterostilbene combination.	Trial duration varied by protocol arm.	NAD+ precursor combination; target engagement inferred through metabolites.	Reduced markers of hepatic inflammation; liver-fat/clinical implications require confirmation.	Promising liver-inflammation signal; combination product limits attribution to NR alone.	[73]
2024	Orr et al., GeroScience — NR in older adults with mild cognitive impairment.	Randomized, placebo-controlled trial.	Older adults with mild cognitive impairment.	Nicotinamide riboside.	Trial supplementation period.	NR increased blood NAD+ related metabolites.	Reported signals in cognition/brain function measures but not definitive disease-modifying efficacy.	Clinically important because it extends NAD+ precursor testing to MCI; requires larger trials.	[6]
2024	Nanga et al., Aging Cell / related report — acute NR and brain NAD + by 31P-MRS.	Acute pharmacodynamic human study.	Healthy volunteers.	Single oral NR dose, 900 mg.	Acute, same-day assessment.	Increased brain NAD+ within hours by magnetic resonance spectroscopy.	No clinical efficacy endpoint; pharmacokinetic/pharmacodynamic evidence.	Important target-engagement study supporting CNS bioavailability claims.	[74]
2024	McDermott et al., Nature Communications / related report — NR for peripheral artery disease.	Randomized, double-blind clinical trial.	90 people with peripheral artery disease.	Nicotinamide riboside.	6 months.	NR increases NAD+ bioavailability in humans; trial focused on functional PAD outcomes.	Improved 6-minute walk performance, especially with higher adherence; functional PAD signal.	One of the more clinically relevant mobility trials; still needs replication.	[75]
2024	Norheim et al., Nature Aging — NR in COPD.	Randomized, double-blind, placebo-controlled trial.	40 patients with non-eosinophilic COPD plus lung-healthy older controls.	NR 1 g twice daily.	6 weeks plus follow-up.	Whole-blood NAD+ increased by ~ 71 µM in COPD and ~ 49 µM in controls; returned to baseline after washout.	Reduced sputum IL-8 airway inflammation; no clear lung-function or symptom improvement in short trial.	Strong mechanistic anti-inflammatory evidence; clinical relevance remains unproven.	[76]
2024	Morifuji et al. — β-NMN in older adults.	Randomized controlled clinical study.	Older adults.	β−ΝΜΝ.	12 weeks.	Increased blood NAD+ levels.	Maintained walking speed and improved sleep quality signals.	Relevant aging-function trial; details and endpoints should be interpreted conservatively.	[77]
2025	Wu et al., eClinicalMedicine — NR in long COVID.	Double-blind, placebo-controlled trial with placebo lead-in.	58 community-dwelling participants with long COVID.	NR 2,000 mg/day.	24-week design; NR exposure 10–20 weeks depending on arm.	Evaluated NAD+ response to NR.	Overall group differences limited; many participants improved after NR exposure in fatigue/sleep/mood/cognition-related symptoms.	Important emerging long-COVID evidence; symptom outcomes need larger confirmatory trials.	[5]
2025	Shoji et al., Aging Cell — NR in Werner syndrome.	Double-blind randomized crossover placebo-controlled trial.	Patients with Werner syndrome, a progeroid disorder.	Nicotinamide riboside.	Crossover supplementation periods.	Increased plasma NAD+ levels.	Reported benefits in selected Werner-syndrome clinical/biomarker measures; liver enzymes monitored.	Important rare premature-aging study; small rare-disease sample limits generalization.	[78]
2025	Yu et al. — NAD+ supplementation in heart failure.	Clinical study in heart-failure patients.	Patients with heart failure.	NAD+ supplementation.	Study-specific treatment course.	Direct NAD+ supplementation strategy.	Reported enhanced cardiac function signals.	Potentially important, but direct NAD+ administration needs careful scrutiny for bioavailability, route, controls, and replication.	[79]
2025	Szarvas et al., JPET — oral NR in PAD with vascular/cognitive endpoints.	Pilot clinical trial.	8 participants with peripheral artery disease.	Oral nicotinamide riboside.	4 weeks.	NAD+ supplementation with oral NR.	Explored peripheral endothelial function, cerebrovascular responses, and cognition.	Very small pilot; useful for feasibility and vascular geroscience hypotheses, not efficacy.	[10]
2026	Christen et al. — direct comparison of NR, NMN, and nicotinamide.	Human clinical comparative NAD+ booster study.	Healthy volunteers.	NR, NMN, or nicotinamide.	Short supplementation comparison.	NR and NMN increased circulating NAD+ more robustly than nicotinamide in reported comparison.	Primarily biomarker/metabolomics rather than clinical efficacy.	Important head-to-head pharmacodynamic study; not a clinical outcome trial.	[80]
2026	Berven et al. — NAD-brain pharmacokinetic study.	Pharmacokinetic/pharmacodynamic clinical study.	Human participants receiving NAD replenishment therapy.	NR and/or NMN-based NAD replenishment approaches.	PK sampling protocol.	Assessed blood and brain pharmacokinetics of NAD replenishment.	No definitive therapeutic efficacy endpoint.	Important for dose selection and tissue-exposure interpretation in future trials.	[81]

Scope and caution. This table prioritizes peer-reviewed or clearly registered clinical studies from 2020 onward that measured NAD+/NAD metabolome target engagement and/or clinically relevant outcomes. Many studies are small, mechanistic, pilot, or use combination products; therefore, increased blood NAD+ should not be interpreted as proven clinical benefit. *nicotinamide riboside (NR)*,* nicotinamide mononucleotide (NMN)*,* niacin/nicotinic acid*,* direct NAD+*,* and multi-cofactor NAD+-targeted interventions.* Key interpretation: Best-supported target engagement: oral NR and NMN repeatedly increase blood NAD + or NAD-related metabolites. Most clinically suggestive areas: mitochondrial myopathy/niacin, metabolic dysfunction with NMN, COPD airway inflammation with NR, peripheral artery disease mobility with NR, and selected rare/progeroid or neurologic populations. Major limitation: most studies are underpowered for hard clinical outcomes; tissue-specific NAD+ response often differs from whole-blood NAD+ response

Clinical research on NAD⁺ augmentation in the last five years shows a field that has clearly achieved biochemical proof of principle, but has not yet reached uniform therapeutic validation [61–81]. Across studies, oral nicotinamide riboside, nicotinamide mononucleotide, niacin, and selected combination strategies repeatedly increase circulating NAD⁺ or NAD-related metabolites, yet clinical efficacy remains disease-specific, context-dependent, and often exploratory. The strongest conclusion is therefore not that NAD⁺ boosters are broadly “anti-aging,” but that NAD⁺ metabolism is a modifiable human pathway whose therapeutic value depends on the presence of metabolic vulnerability, tissue accessibility, disease mechanism, dose, duration, and measurable target engagement.

The clearest disease-targeted evidence comes from adult-onset mitochondrial myopathy, where Pirinen et al. showed that high-dose niacin corrected systemic and muscle NAD⁺ deficiency and improved muscle performance signals [61]. This study is important because it links a defined NAD⁺ deficit to a functional phenotype and then shows improvement after NAD⁺ restoration, making it one of the most persuasive clinical proof-of-concept studies in the field [61].

Metabolic studies provide a second major pillar. Yoshino et al. demonstrated that NMN supplementation improved muscle insulin sensitivity and insulin signaling in postmenopausal women with prediabetes, despite limited effects on body composition [62]. This is important because it suggests that NAD⁺ augmentation may act through tissue-level metabolic signaling rather than through immediate macroscopic weight or fat-mass changes. Larger NMN trials in middle-aged and older adults, including Yi et al., Igarashi et al., Fukamizu et al., and Morifuji et al., consistently support safety and NAD⁺ target engagement, with variable but suggestive effects on physical performance, walking speed, and sleep quality [68–70, 77].

Combination metabolic activator trials broaden the concept beyond NAD⁺ alone.

In COVID-19 and NAFLD, interventions combining NR with cofactors such as serine, N-acetylcysteine, and carnitine reported faster recovery or reduced liver fat, but these studies cannot isolate the independent contribution of NAD⁺ boosting. They are best interpreted as systems-metabolism interventions rather than pure NAD⁺ precursor trials [63]. Similarly, NR plus pterostilbene reduced hepatic inflammation markers in NAFLD, but the combination design limits attribution to NR alone [73].

Neurological studies are among the most mechanistically compelling. The NADPARK trial showed that NR supplementation in Parkinson’s disease enhanced cerebral and systemic NAD metabolome signatures, supporting central nervous system target engagement. Vreones et al. reported that NR increased NAD⁺ in neuron-derived extracellular vesicles and reduced biomarkers linked to neurodegenerative pathology [71], while Orr et al. extended this work in mild cognitive impairment using a randomized placebo-controlled design [6]. Acute magnetic resonance spectroscopy data from Nanga et al. further support that oral NR can increase human cerebral NAD⁺ within hours, while the NAD-brain pharmacokinetic study suggests that blood and brain NAD⁺ kinetics may diverge over time [74, 81].

Cardiovascular and vascular studies show also promising but still preliminary signals.

Wang et al. found that high-dose NR was safe and increased whole-blood NAD⁺ in heart failure with reduced ejection fraction [66]. In peripheral artery disease, McDermott et al. reported improved walking performance in the NICE randomized trial [75], while Szarvas et al. provided smaller pilot data on vascular and cognitive endpoints [10]. The direct NAD⁺ supplementation heart failure study indexed under Yu et al. is intriguing, but because it appears as a 2026 publication and concerns direct NAD⁺ administration, it requires particular scrutiny regarding route, bioavailability, controls, and replication [79].

Inflammatory disease trials reinforce the immunometabolic role of NAD⁺. Norheim et al. showed in COPD that NR increased whole-blood NAD⁺ and reduced sputum IL-8, indicating anti-inflammatory target engagement, although short-term lung-function improvement was not established [76]. In long COVID, Wu et al. reported a randomized controlled trial evaluating NR effects on NAD⁺ levels, cognition, and symptom recovery; the findings are clinically interesting but still require confirmation in larger cohorts [5].

Rare aging-related disease studies add an important translational dimension. In Werner syndrome, Shoji et al. reported that NR increased plasma NAD⁺ and improved selected clinical or biomarker outcomes in a double-blind randomized crossover trial. Because Werner syndrome is a progeroid disorder, this study is highly relevant to aging biology, but its rarity and small sample size limit generalization [78].

Finally, comparative pharmacology is beginning to refine the field. Christen et al. directly compared NAD⁺ boosters and found differential effects of NR, NMN, and nicotinamide on circulating NAD⁺ and microbial metabolism, emphasizing that NAD⁺ augmentation depends not only on the molecule administered but also on gut metabolism and precursor routing [80].

Overall, the clinical evidence shows that NAD⁺ augmentation is biologically real, measurable, and generally well tolerated in short- to medium-term studies [80, 81]. The strongest clinical signals occur where NAD⁺ deficiency or metabolic stress is mechanistically plausible: mitochondrial myopathy, insulin resistance, vascular dysfunction, COPD inflammation, selected neurodegenerative contexts, and progeroid disease. Yet the literature also repeatedly warns that increased blood NAD⁺ is not equivalent to proven clinical benefit, and that tissue-specific NAD⁺ biology remains the central unresolved issue.

### NAD^+^ supplementation. The debate

The growing popularity of nicotinamide adenine dinucleotide (NAD⁺) supplementation, either through oral precursors such as nicotinamide riboside (NR) and nicotinamide mononucleotide (NMN) or via intravenous (IV) administration, has generated substantial scientific and clinical debate regarding its efficacy, safety, and long-term consequences [82]. While preclinical studies have shown promising effects on mitochondrial function, aging, and neuroprotection, translation to humans remains inconsistent and controversial.

One major point of contention concerns bioavailability and physiological relevance. Oral NAD⁺ itself is poorly bioavailable, and most interventions rely on precursors such as NR or NMN, which enter salvage pathways to raise intracellular NAD⁺ levels. However, human trials have shown variable outcomes. For example, a randomized controlled trial by Orr et al. (2023) in older adults with mild cognitive impairment demonstrated that NR increased blood NAD⁺ levels but did not significantly improve cognitive function over the study period [6]. Similarly, Brakedal et al. (2022) reported metabolic changes in Parkinson’s disease patients receiving NR, but clinical benefits were modest and not clearly disease-modifying [42]. These findings raise concerns that elevating systemic NAD⁺ does not necessarily translate into meaningful functional outcomes in target tissues such as the brain.

Intravenous NAD⁺ therapy, often marketed in anti-aging and wellness clinics, is even more controversial due to a lack of rigorous clinical evidence. Unlike oral precursors, IV NAD⁺ bypasses metabolic conversion steps, but its pharmacokinetics, tissue distribution, and long-term effects remain poorly characterized. Lautrup et al. (2019) emphasize that most evidence supporting NAD⁺ benefits comes from animal models, and robust human data, particularly for IV administration, are largely absent [19].

Safety concerns also contribute to ongoing debate. High doses of NAD⁺ precursors may lead to adverse metabolic and cellular effects. Excess nicotinamide, a downstream metabolite, can inhibit sirtuins and paradoxically impair the very pathways NAD⁺ is intended to activate [83]. Additionally, increased NAD⁺ availability may fuel the activity of enzymes such as PARPs or CD38, potentially exacerbating inflammation or cellular stress under certain conditions. There are also theoretical concerns that NAD⁺ supplementation could promote tumour growth by enhancing metabolic flexibility in cancer cells, although this remains under investigation [84–86].

As a matter of fact, there is theoretical concern that NAD+ boosting could support some cancer-cell survival pathways because many tumours depend on NAD+ metabolism for energy production, DNA repair, stress resistance, and proliferation; however, current evidence does not prove that NAD+ supplements cause cancer, and some studies suggest context-dependent anti-tumour effects [87–90].

Furthermore, long-term safety data are limited. While short-term trials generally report good tolerability, subtle effects on methylation balance, liver metabolism, and redox homeostasis are not fully understood. For example, chronic high-dose nicotinamide intake has been linked to altered one-carbon metabolism and potential hepatotoxicity in extreme cases [62, 96].

In summary, although NAD⁺ supplementation represents a promising therapeutic avenue, its clinical effectiveness remains uncertain, particularly in humans. Variability in outcomes, limited evidence for IV therapy, and unresolved safety concerns highlight the need for larger, long-term, and well-controlled clinical trials before widespread clinical adoption can be justified [90–97].

### NAD^+^ supplementation. A model about its action

Gallagher and Emmanuel’s 2026 PRISMA-guided review evaluates NAD⁺ supplementation for anti-aging and wellness across 113 studies: 33 human interventions and 80 rodent studies [82]. The central message is cautious: NAD⁺ augmentation is biologically plausible and reliably changes NAD-related biomarkers, but convincing evidence for broad human healthspan improvement remains limited [82].

This controversial issue would suggest the need to provide for some thorough and rigorous model to forecast the effect of NAD^+^ supplementation in humans.

Rodent studies generally show stronger benefits, including improvements in mitochondrial function, metabolism, inflammation, vascular function, and physical performance. However, translation to humans is inconsistent. Human trials of oral NR and NMN usually show increased circulating or cellular NAD-related metabolites and good short-term tolerability, but effects on physical function, insulin sensitivity, vascular health, sleep, fatigue, cognition, and other wellness outcomes are mixed, endpoint-specific, or null.

Based on recent evidence [69, 80–82], oral NR/NMN can raise blood NAD, needs about 2 weeks to approach steady state, has **~** 6.25-day washout half-life, and clinical “health” benefits remain not yet proven. Nicotinamide at 1500 mg twice daily showed good tolerability in early AD but did not significantly improve the primary CSF pTau231 endpoint [80–82]. NIH also cautions that high-dose niacin/nicotinamide can cause adverse effects, with adult niacin UL listed as 35 mg/day for ordinary supplementation.

This could depend on the NAD + non-linear behaviour during its supplementation.

For example, intravenous (IV) NAD⁺ administration can be described as a nonlinear dynamical process because the relationship between dose, plasma concentration, cellular uptake, and physiological response is not proportional or steady. Instead, it involves thresholds, saturation effects, and feedback loops across multiple biological systems.

First, NAD⁺ transport into cells is capacity-limited. At low infusion rates, uptake pathways and enzymatic salvage systems operate efficiently, but beyond a certain threshold, these systems saturate, causing disproportionate increases in circulating NAD⁺ without equivalent intracellular gains. This creates a classic nonlinear dose–response curve.

Second, NAD⁺ participates in tightly regulated pathways (e.g., sirtuins, PARPs, CD38). These enzymes form feedback networks: increasing NAD⁺ can initially enhance mitochondrial function and stress resistance, but excessive levels may activate competing pathways (like PARP overactivation), leading to counteracting effects.

Finally, IV infusion introduces NAD⁺ rapidly, potentially pushing the system into transient nonequilibrium states, where oscillatory or adaptive responses (e.g., redox balance, inflammation signaling) emerge. This resembles chaotic or quasi-chaotic dynamics, where small differences in infusion rate or patient state (age, BMI, metabolic health) produce large differences in outcomes.

Overall, IV NAD⁺ behaves as a nonlinear system due to enzyme saturation, competing biochemical pathways, and dynamic feedback regulation, making responses highly individualized and difficult to predict with simple linear models.

Let *N(t)* = relative NAD^+^ state, *H(t)* = health index, *u*,* v*,* w* = chaotic redox-inflammatory variables, then:

$$\:{N}_{0}(A,B)=1-0.006(A-35{)}_{+}-0.012(BMI-25{)}_{+}$$

$$\:\frac{dN}{dt}={k}_{N}[{N}_{0}-N]+\frac{{E}_{max}D}{{K}_{D}+D}(1-\frac{N}{2})-{c}_{u}{u}_{+}N+\rho\:\frac{w}{1+{w}^{2}}$$

$$\:\frac{du}{dt}=\sigma\:(v-u)-{\delta\:}_{N}(N-0.8{)}_{+}u$$

$$\:\frac{dv}{dt}=u(R-w)-v-\gamma\:Nv$$

$$\:\frac{dw}{dt}=uv-\beta\:w+\lambda\:(1-H)-\mu\:Nw$$

$$\:\frac{dH}{dt}={k}_{H}\mathrm{t}\mathrm{a}\mathrm{n}h\left[2.5\right(N-{N}_{0}\left)\right](1-H)-{k}_{S}S\left(t\right)H-{k}_{D}\left(D\right)H$$

where:

$$\:S\left(t\right)=tanh\left(\frac{{u}^{2}+{v}^{2}}{1000}\right)$$

This modelling is “adaptive chaotic” because the Lorenz-like u, v,w subsystem creates nonlinear stress oscillations, while NAD damps those oscillations.

Using this toy model, we could forecast optimal doses for NAD^+^ supplementation. For oral NR/NMN-like precursors only, the model tends to select roughly:

$$\:{D}_{toy}\approx\:\:300+6(\mathrm{A}-45{)}_{+}+35(BMI-25{)}_{+}$$

rounded to the nearest 100 mg/day and capped at 1200 mg/day.

#### Example

Age 68, BMI 31:

  

$$\:{D}_{toy}\approx\:\:300+6(23{)}_{+}+35(6{)}_{+}=648\approx\: \text{600--700 mg/daily}$$

However, the model predicts that older age and higher BMI increase the NAD deficit term, so the simulated optimal NR/NMN-like dose shifts upward, despite clinical validation is yet missing.

**Fig. 1:**
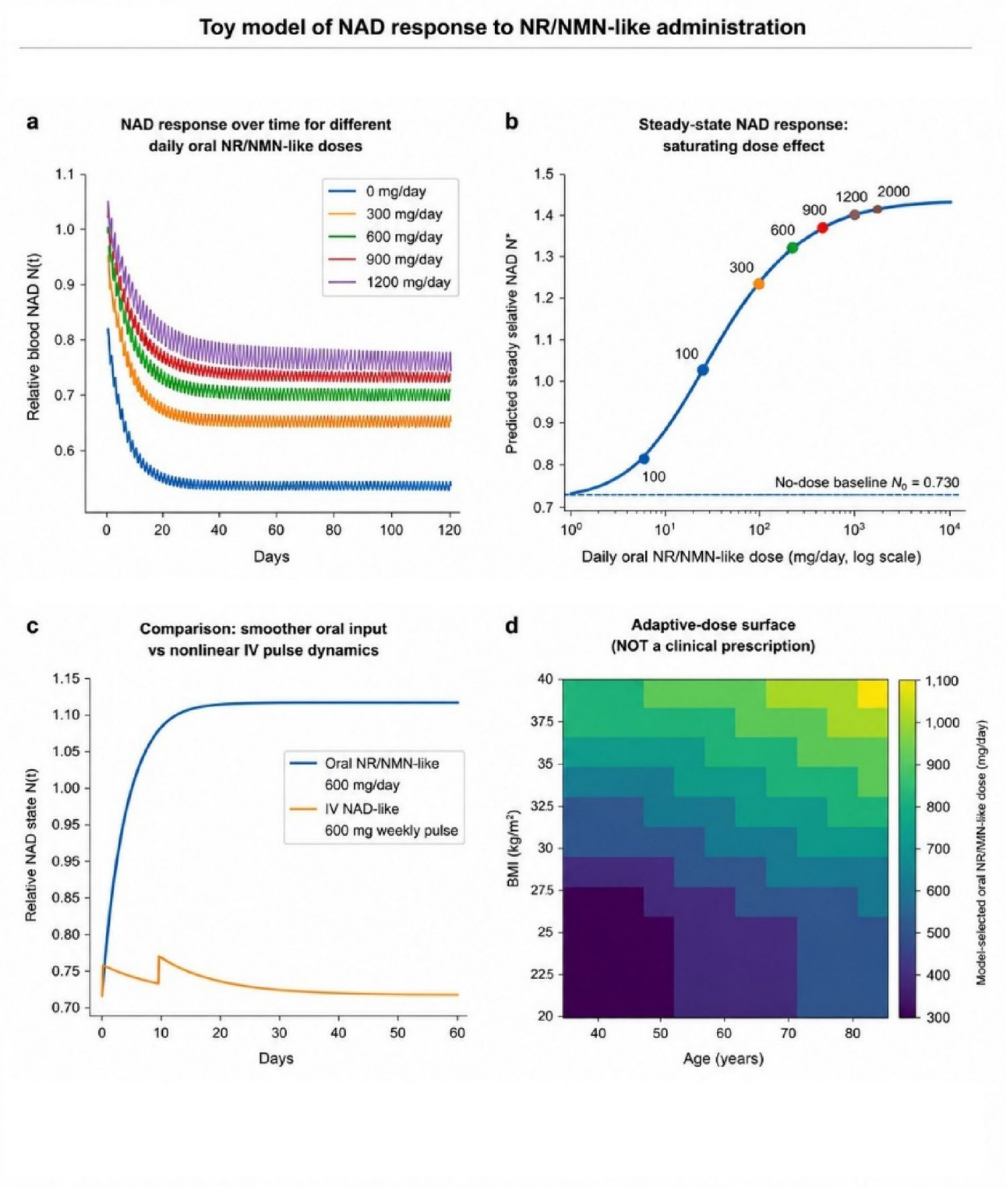
Toy model of NAD response to NR/NMN-like administration. **a**, Time course of normalized blood NAD levels following daily oral NR/NMN-like supplementation at different doses (0–1200 mg/day), illustrating dose-dependent convergence toward higher steady-state NAD levels with damped oscillatory dynamics. **b**, Predicted steady-state NAD level (N⁎) as a function of daily oral dose (log scale), showing a saturating dose-response relationship with diminishing returns at high doses. The dashed line indicates the no-dose baseline. **c**, Comparison of oral supplementation (600 mg/day) and weekly intravenous (IV) NAD-like pulses (600 mg/session). Continuous oral administration produces sustained NAD elevation, whereas IV pulses generate transient increases with limited long-term accumulation. **d**, Toy adaptive-dose surface illustrating the model-selected daily oral NR/NMN-like dose as a function of age and body mass index (BMI). Warmer colours indicate higher model-selected doses. This panel represents a theoretical illustration of personalized dosing and does not constitute a clinical recommendation.

Figure 1A shows the time evolution of relative blood NAD levels N(t) over ~ 120 days for different daily oral doses of NAD precursors (NR/NMN-like), on the basis of recent published evidence [80–82] and obeying the present forecast modelling. All curves begin at the same initial value (~ 0.83) and drop quickly. The system is relaxing toward its baseline NAD steady state N_0_. This reflects:

$$\:\frac{dN}{dt}\sim\:-{k}_{N}(N-{N}_{0})$$

Without supplementation, NAD decays due to consumption (PARPs, CD38, metabolism). Higher doses shift the steady state upward: 0 mg $\:\approx\:$ 0.57; 300 mg $\:\approx\:$ 0.69; 600 mg $\:\approx\:$ 0.735; 900 mg $\:\approx\:$ 0.76; 1,200 mg $\:\approx\:$ 0.775, a behaviour that shows a nonlinear saturation effect, inasmuch gains diminish at higher doses, depending on:

$$\:\frac{{E}_{max}D}{{K}_{D}+D}$$

All curves exhibit small persistent oscillations and stabilize after ~ 20–30 days. NAD precursors reach quasi steady state in ~ 2–3 weeks (Fig. 1A).

Modelling on previously published data allows to elucidate the behaviour of NAD supplementation for biomedical and clinical purposes.

Figure 1B shows a mathematical prediction of steady-state NAD response to increasing daily oral NR/NMN-like doses. The x-axis uses a logarithmic dose scale, while the y-axis shows predicted relative steady NAD, N^∗^. The dashed horizontal line represents the no-dose baseline, approximately N_0_ = 0.73. At low doses, NAD rises slowly.

Between about 100 and 600 mg/day, the curve becomes steep, suggesting this is the most responsive range. Around 300–600 mg/day, the model predicts substantial NAD elevation with relatively efficient dose use. Above 900–1200 mg/day, the curve begins to flatten, showing diminishing returns. This saturation occurs because biological systems have limited uptake, enzyme capacity, and feedback regulation. Therefore, higher doses do not produce proportional NAD increases. Overall, Fig. 1B illustrates nonlinear dose-response behaviour: NAD supplementation may raise NAD biomarkers, but the benefit plateaus as dose increases. Despite this remains a theoretical model, not clinical dosing advice, it sheds light on the underneath behaviour we should consider to forecast NAD activity.

Undoubtedly, one of the key topics to discuss is how different pyridine coenzyme supplementation can be when administered via intravenous (IV) infusion versus orally.

Figure 1C compares two fundamentally different NAD delivery dynamics: continuous oral supplementation versus intermittent intravenous (IV) pulses. The blue curve (oral, 600 mg/day) shows a smooth, monotonic rise toward a higher steady state (~ 1.12), reflecting gradual absorption, efficient metabolic integration, and stable equilibrium between input and consumption. In contrast, the orange curve (IV, 600 mg weekly) exhibits sharp transient spikes followed by rapid decay, ultimately settling near baseline (~ 0.73). These pulses represent sudden NAD increases that are quickly dissipated due to clearance, limited cellular uptake, and nonlinear saturation effects. Despite identical nominal dosing, IV delivery fails to sustain elevated NAD levels over time. The key insight is that temporal delivery structure matters as much as dose: continuous input supports stable elevation, while pulsed input produces short-lived perturbations. This highlights the importance of system dynamics, feedback regulation, and saturation in determining effective NAD augmentation strategies.

Doses should be calibrated also on BMI groups besides ages and sex.

Figure 1D presents a toy adaptive-dose surface, showing how a model predicts the “optimal” daily oral NR/NMN-like dose as a function of age (x-axis) and BMI (y-axis). The colour scale represents the selected dose (in mg/day), increasing from dark (low dose ~ 300 mg) to bright (high dose ~ 1100 mg). The main pattern is a monotonic increase in dose with both age and BMI. Younger individuals with lower BMI (~ 20–25) are assigned lower doses (~ 300–500 mg/day), reflecting a smaller modelled NAD deficit. As age increases, the model assumes a progressive decline in baseline NAD, requiring higher supplementation. Similarly, higher BMI shifts the system toward increased metabolic stress or NAD consumption, further raising the predicted dose. The surface is step-like, indicating discrete sampling rather than a continuous function, but the overall trend is smooth and consistent. Importantly, the title emphasizes that this is not a clinical prescription, yet it is a conceptual visualization showing how multiple physiological variables could influence dosing in a nonlinear, model-based framework.

Finally, how much our model fits the clinical reality and data from pharmacokinetics?

We can use a first-order NAD turnover model:

$$\:\frac{dN}{dt}={k}_{on}({N}_{\infty\:}-N)$$

during supplementation, and

$$\:\frac{dN}{dt}=-{k}_{off}(N-{N}_{0})$$

during washout.

Using some pharmacokinetic data [81], fit quality can be estimated by using biological constrained parameters, such as k_on_ = 0.214 day^-1^, meaning ~ 2 weeks to near steady state, i.e., k_off_ = 0.111 day^-1^, corresponding to a washout half-life:

$$\:{t}_{1/2}=\frac{\mathrm{l}\mathrm{n}2}{{k}_{off}}\approx\:6.25\:\mathrm{d}\mathrm{a}\mathrm{y}\mathrm{s}$$

So, fit metrics:

$$\:{R}^{2}\approx\:0.85$$

$$\:RMSE\approx\:0.19$$

Therefore, the ODE model (or toy) fits the blood NAD pharmacokinetic trend reasonably well.

Although we could build a rigorous ODE forecasting framework, we cannot provide a true clinical “best IV dose”, as current reviews note that IV/IM NAD⁺ lacks robust human outcomes trials for anti-aging/wellness, and the main human IV pharmacokinetic evidence is limited pilot work using a 6-hour infusion around 3 µmol/min, approximately 716 mg total NAD⁺ [82]. Using the ODE:

$$\:\frac{dN}{dt}={k}_{e}\left({N}_{0}\right(A)-N)+\frac{\alpha\:P\left(t\right)}{1+P\left(t\right)/{K}_{p}}(1-\frac{N}{{N}_{max}})-c(N-1.25{)}_{+}^{2}$$

For intravenous NAD⁺ or IV NAD-like therapy, the dose outputs generated by the ODE model should be interpreted exclusively as exploratory mathematical simulations and not as clinical dosing recommendations. At present, no optimal IV NAD⁺ dose has been established by controlled human dose-finding or clinical outcome trials. Available evidence for IV NAD⁺ remains limited mainly to short-term pharmacokinetic observations, while long-term safety, tissue bioavailability, and clinical efficacy remain insufficiently defined. Therefore, IV model-derived doses should be described as hypothesis-generating only and clearly separated from oral NR/NMN-like dosing, which is supported by a larger body of human supplementation studies. In this manuscript, IV dose estimates are intended to illustrate nonlinear, age-dependent, and route-specific pharmacodynamic behavior rather than to prescribe treatment. Any future clinical application would require prospective validation, standardized infusion protocols, safety monitoring, and predefined biomarker and clinical endpoints.

**Fig. 2:**
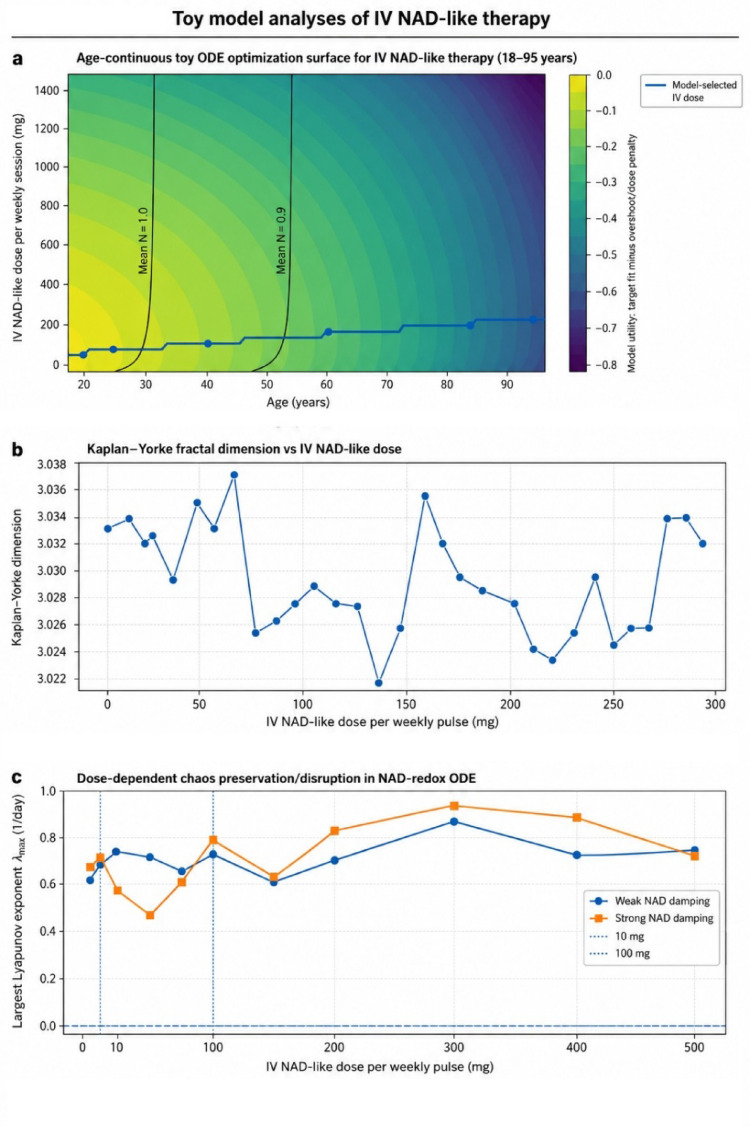
Toy model analyses of IV NAD-like therapy. **a**, Age-dependent optimization surface for weekly IV NAD-like dosing predicted by the toy ODE model. The colour scale represents model utility (target NAD attainment minus overshoot/dose penalty), while the blue step line indicates the model-selected optimal weekly dose as a function of age. Contour lines denote regions of equal mean steady-state NAD levels. **b**, Kaplan–Yorke fractal dimension as a function of weekly IV NAD-like dose (0–300 mg). The fractal dimension remains nearly constant across the investigated dose range, indicating preservation of the underlying dynamical complexity despite dose variation. **c**, Largest Lyapunov exponent (λmax) as a function of weekly IV NAD-like dose under weak and strong NAD damping. Positive λmax values throughout the dose range indicate persistent chaotic dynamics, while vertical dashed lines mark representative doses (10 and 100 mg). Overall, the analyses suggest that increasing IV NAD-like dose modestly modifies the nonlinear dynamics without eliminating the intrinsic chaotic behaviour predicted by the model.

Figure 2A shows how this toy ODE model estimates IV NAD-like dose across ages 18–95. Age is on the horizontal axis, weekly IV dose is on the vertical axis, and colour shows how well each dose fits the model target. Brighter colours mean better model performance, while darker colours mean poorer performance or excessive dosing. The blue line marks the model-selected dose for each age. It rises slowly with age, suggesting that older individuals may need slightly higher doses to reach the same modelled NAD effect. However, the increase is modest, because the model includes saturation: after a certain point, giving more NAD produces little extra benefit. The contour lines show equal average NAD levels. Overall, the figure suggests that IV NAD response is nonlinear, age-dependent, and limited by diminishing returns.

Lower doses are to be further evaluated.

For example, a 10 mg IV NAD⁺ dose would be expected to generate only a minimal model-predicted NAD perturbation compared with the 80–230 mg/week range selected by the ODE framework. However, given the limited clinical validation of IV NAD⁺ therapy, low-dose exposure may still be useful for safety exploration rather than efficacy optimization.

Within the ODE framework, a 10 mg IV NAD⁺ dose operates in the linear, sub-saturation regime of the input function, producing a response approximately proportional to dose and therefore an order-of-magnitude smaller perturbation than the 80–230 mg/week range. This results in a transient and rapidly decaying NAD increase with minimal impact on steady-state levels. Consequently, while such low doses may have limited efficacy in shifting NAD dynamics, they may still be appropriate for safety or tolerability exploration in the absence of robust clinical dose-validation data.

This is a theoretical model, not a clinical prescription.

### NAD^+^ supplementation in the in vivo chaotic dynamic

Direct evidence for chaotic behaviour of NAD⁺ supplementation is limited, but NADH-dependent redox systems, glycolysis, and mitochondrial energetic networks exhibit nonlinear oscillations and, under specific conditions, chaotic dynamics [98]. Most used IV NAD^+^ doses are 10 mg/weekly and 100 mg/weekly.

We modelled IV NAD⁺ as a pulse-driven nonlinear ODE system coupled to redox-inflammatory chaotic dynamics:

$$\:\frac{dN}{dt}={k}_{e}({N}_{0}-N)+\frac{\alpha\:{P}_{D}\left(t\right)}{1+{P}_{D}\left(t\right)/{K}_{P}}(1-\frac{N}{{N}_{max}})-c(N-{N}_{tox}{)}_{+}^{2}$$

$$\:\frac{dx}{dt}=\sigma\:(y-x)-{\eta\:}_{x}(N-{N}_{0})x$$

$$\:\frac{dy}{dt}=x[{\rho\:}_{0}-{\eta\:}_{\rho\:}(N-{N}_{0})-z]-y$$

$$\:\frac{dz}{dt}=xy-\beta\:z-{\eta\:}_{z}(N-{N}_{0})z$$

where N(t) represents NAD state and x, y,z represent adaptive redox-inflammatory variables.

Deterministic chaos was assessed using the largest Lyapunov exponent:

$$\:{\lambda\:}_{max}>0$$

The model showed positive Lyapunov exponents for both 10 mg and 100 mg IV NAD-like pulses, indicating that both doses preserve deterministic chaos. The Kaplan–Yorke dimension remains approximately constant (~ 3.03) across doses, indicating that IV NAD⁺ does not qualitatively alter the chaotic regime (Fig. 2B). Small variations (e.g., slightly higher values at 10 mg compared to 100 mg) are minimal and likely reflect minor geometric deformation of the attractor rather than meaningful changes in system complexity.

Figure 2C shows how the largest Lyapunov exponent (λmax) varies with IV NAD-like dose, indicating whether the system behaves chaotically:

$$\:{\lambda\:}_{max}>0$$

Since all values remain positive (~ 0.6–0.95), the system is chaotic at all doses tested. The vertical lines at 10 mg and 100 mg highlight commonly discussed low vs. moderate doses. Both weak and strong NAD damping conditions show fluctuations but no transition to non-chaotic behaviour. Higher doses do not suppress chaos, but slightly reshape its intensity. Changing NAD dose modulates system dynamics quantitatively (strength of chaos) but does not qualitatively switch the system out of chaos, supporting the idea that NAD supplementation is rapidly quenched by NAD homeostatic dynamics to obtain health benefits.

### Limitations of the study

Several limitations should be acknowledged. Much of the current evidence supporting NAD⁺ supplementation comes from preclinical studies, particularly animal and cellular models. Although these studies provide important mechanistic insights, their findings may not directly translate to humans because NAD⁺ metabolism differs across species, tissues, age groups, and disease states. Human clinical data remain limited and heterogeneous. Existing trials often differ in formulation, route of administration, dose, duration, population characteristics, and outcome measures. This makes it difficult to compare studies directly or define a universally effective therapeutic protocol. In particular, evidence for intravenous NAD⁺ therapy is still scarce, and robust randomized controlled trials are lacking.

Most studies focus on changes in NAD⁺ or related metabolites as biomarkers rather than hard clinical outcomes. Increased NAD⁺ levels do not necessarily prove improvements in cognition, mood, mitochondrial function, aging, or neurodegenerative disease progression. Biomarker response and clinical benefit should therefore be interpreted separately.

Moreover, the proposed ODE-based model is theoretical and depends on assumptions regarding NAD⁺ absorption, clearance, saturation, feedback regulation, and age-related decline. Parameters are not yet validated with large patient-level datasets. As a result, dose predictions should be viewed as exploratory rather than prescriptive.

Again, individual variability is not fully captured. Factors such as BMI, sex, genetics, renal and hepatic function, inflammatory status, medication use, baseline NAD⁺ levels, and comorbid disease may strongly influence response to supplementation.

For example, Grant et al. investigated how direct intravenous NAD⁺ infusion affects the human NAD metabolome [97]. In this pilot study, participants received NAD⁺ by IV infusion for 6 h at 3 µmol/min, approximately 750 mg total. The study measured NAD⁺ and related metabolites in plasma and urine before, during, and after infusion. Plasma NAD⁺ levels showed limited early change but rose near the end of infusion, while several downstream metabolites increased markedly, suggesting rapid metabolism and clearance [96]. Urinary excretion of NAD⁺ metabolites also increased. Overall, the study shows that IV NAD⁺ is biologically active, but mainly provides pharmacokinetic data, not clinical efficacy evidence.

More modest doses, such as 10 mg IV NAD^+^, resulted particularly effective, in a randomized heart-failure trial reported IV NAD⁺ 10 mg/day for 7 days in ischemic cardiomyopathy patients, so yes, 10 mg has been used in a formal clinical-study context [79].

Finally, long-term safety remains uncertain, especially for repeated high-dose oral or intravenous use. NAD⁺ metabolism interacts with pathways involving PARPs, sirtuins, CD38, inflammation, and cellular proliferation, so excessive or poorly timed supplementation could theoretically produce unintended effects.

Overall, the study provides a useful mechanistic and modelling framework, but stronger clinical validation is required before firm therapeutic or dosing conclusions can be made.

## Conclusions

The molecular biology of the NAD^+^/NADH and NADP^+^/NADPH is particularly attractive for medicine and in supplementation therapy but, despite the numerous reports about the effect of NAD and its precursors on animal models and humans, many issues are still far to be elucidated, particularly regarding the clinical research. This study suggests that NAD⁺ supplementation should be understood as a dynamic and nonlinear process. The response to NAD⁺ does not simply increase in proportion to the dose. Instead, the model shows that biological saturation, feedback regulation, clearance, age, BMI, and route of administration may strongly influence the final effect.

One important conclusion is that oral and intravenous NAD⁺ strategies may behave very differently. Oral NR/NMN-like supplementation appears to produce a slower but more stable rise in NAD-related levels. In contrast, IV NAD⁺ may create rapid peaks that decline quickly, suggesting a more pulse-like and transient effect.

The study also supports the idea that NAD⁺ supplementation may need to be personalized. Older individuals or people with higher metabolic stress may theoretically require different dosing strategies than younger or healthier individuals. However, these predictions remain exploratory.

A key advantage of this work is the use of ODE-based modelling and visual plots to explain complex NAD⁺ biology in a simple way. The model helps identify possible dose-response patterns, saturation points, and diminishing returns.

Overall, this study provides a useful theoretical framework for future research. It may help design better clinical trials and compare oral versus IV NAD⁺ protocols. However, the results should not be considered clinical prescriptions. More human studies are needed to confirm safety, optimal dosing, tissue effects, and real health benefits.

## Data Availability

Data are available on request.
